# CD8^+^ T-Cells as Immune Regulators of Multiple Sclerosis

**DOI:** 10.3389/fimmu.2015.00619

**Published:** 2015-12-10

**Authors:** Sushmita Sinha, Alexander W. Boyden, Farah R. Itani, Michael P. Crawford, Nitin J. Karandikar

**Affiliations:** ^1^Department of Pathology, University of Iowa, Iowa City, IA, USA

**Keywords:** CD8, multiple sclerosis, EAE, T-cells, immune regulation

## Abstract

The vast majority of studies regarding the immune basis of MS (and its animal model, EAE) have largely focused on CD4^+^ T-cells as mediators and regulators of disease. Interestingly, CD8^+^ T-cells represent the predominant T-cell population in human MS lesions and are oligoclonally expanded at the site of pathology. However, their role in the autoimmune pathologic process has been both understudied and controversial. Several animal models and MS patient studies support a pathogenic role for CNS-specific CD8^+^ T-cells, whereas we and others have demonstrated a regulatory role for these cells in disease. In this review, we describe studies that have investigated the role of CD8^+^ T-cells in MS and EAE, presenting evidence for both pathogenic and regulatory functions. In our studies, we have shown that cytotoxic/suppressor CD8^+^ T-cells are CNS antigen-specific, MHC class I-restricted, IFNγ- and perforin-dependent, and are able to inhibit disease. The clinical relevance for CD8^+^ T-cell suppressive function is best described by a lack of their function during MS relapse, and importantly, restoration of their suppressive function during quiescence. Furthermore, CD8^+^ T-cells with immunosuppressive functions can be therapeutically induced in MS patients by glatiramer acetate (GA) treatment. Unlike CNS-specific CD8^+^ T-cells, these immunosuppressive GA-induced CD8^+^ T-cells appear to be HLA-E restricted. These studies have provided greater fundamental insight into the role of autoreactive as well as therapeutically induced CD8^+^ T-cells in disease amelioration. The clinical implications for these findings are immense and we propose that this natural process can be harnessed toward the development of an effective immunotherapeutic strategy.

## Introduction

Studies addressing the immunobiology of multiple sclerosis (MS) and its animal model experimental autoimmune encephalomyelitis (EAE) have focused on CD4^+^ T-cells as the main orchestrators of pathogenesis and regulation. CD8^+^ T-cells are the most abundant T-cells in CNS lesions of MS patients (1) and exhibit oligoclonal expansion (2–4). This indicates an important role for these cells in the target organ. However, the functional nature of these cells during disease and its treatment is unclear and somewhat controversial. There are abundant CNS-specific (5, 6) and therapeutically induced CD8^+^ T-cell responses in MS patients (5–8). Recent studies suggest that certain MHC class I alleles can be associated with genetic risk or protection in MS (9–11). Functional roles for some of these MHC class I molecules have been tested in the EAE models. 2D1-TCR humanized transgenic mice, expressing MS risk variant HLA-A3 together with TCR that recognizes myelin proteolipid protein (PLP), develope spontaneous EAE in only 4% of mice and mild EAE early on when immunized with PLP peptide. A quarter of these mice went on to develop a severe disease course with 2D1^+^-TCR^+^–CD8^+^ T-cells present in the CNS of these mice, suggesting a pathogenic role for HLA-A3-restricted myelin-specific CD8_+_ T-cells (12). However, introduction of HLA-A2 alleles in the same model completely abrogates spontaneous and induced EAE, providing evidence for the protective role for HLA-A2-restricted CD8^+^ T-cells (12). We are only beginning to understand these responses and here attempt to provide an overview of such studies. We will summarize the evidence for both pathogenic and regulatory functions of CD8^+^ T-cells in MS and EAE. We will provide an overview of the various cellular and molecular interactions that mediate the role of these cells and develop a model for such functions during disease.

## Pathogenic Role for CD8^+^ T-Cells In EAE

Much of the focus regarding the pathogenesis of EAE has revolved primarily around myelin-specific CD4^+^ T-cells. Adoptive transfer of CD4^+^ T-cells isolated from myelin antigen-primed animals is sufficient to induce disease. This observation partly facilitated the overall ignorance surrounding CD8^+^ T-cells and their potential contribution to disease. A pathogenic role first became evident when a CD8^+^ T-cell-mediated model of EAE was developed using the self-protein myelin basic protein (MBP) (13). In attempts to prime an MHC class I-restricted T-cell response, C3H.Fej, and C3H MBP-deficient shiverer mice were infected with MBP-expressing vaccinia. CD8^+^ T-cell lines specific for MBP_79–87_ drove pathogenesis and demyelination when transferred into wildtype (WT) C3H recipients. Mice developed neurological symptoms including ataxia, spasticity, and lost weight when compared to control animals that received vaccinia-specific CD8^+^ T-cells. Histologically, perivascular cuffs composed primarily of lymphocytes and macrophages were detected in the brain but not in the spinal cord. IFNγ was found to play an important role in mediating MBP-specific CD8^+^ T-cell-driven disease, as its neutralization reduced severity. The break of peripheral tolerance following viral infection was also shown to induce CD8^+^ T-cell-mediated CNS autoimmunity (14). In this report, dual TCR-expressing CD8^+^ T-cells recognizing both viral antigen and MBP triggered disease. Following viral infection, CD8^+^ T-cells, macrophages, and activated microglia infiltrated both the brain and spinal cord. Clinically, mice lost weight and exhibited symptoms of ataxia, impaired mobility, and tail weakness.

CD8^+^ T-cell-mediated EAE has also been induced in C57BL/6 (B6) mice through transfer of myelin oligodendrocyte glycoprotein (MOG)-specific CD8^+^ T-cells (15). MOG-specific CD8^+^ T-cells isolated from mice immunized with MOG_35–55_ peptide were encephalitogenic, and transferred severe paralytic disease to B6 mice. One caveat to this study is that cells were nylon wool-enriched, calling purity into question. Disease was transferred using <1e6 MOG_35–55_ CD8^+^ T-cells and resulted in more severe EAE compared to active immunization. Transferred cells could be re-isolated 6–8 months later, possibly due to additional IL-2 stimulus. How these cells induced pathology was not investigated.

A separate group identified MOG_37–46_-specific CD8^+^ T-cells as autoaggressive effectors (16). In this system, MOG-specific CD8^+^ T-cells were generated following immunization. Restimulation with antigen and IL-2 readily yielded IFNγ from these cells, but not TGFβ or IL-10. These cells, which were found to be H-2D^b^-restricted, could induce EAE when transferred into SCID or naïve WT B6 recipients. MOG_37–46_ elicited the best IFNγ response from MOG_35–55_-primed lymph node cells, although bound MHC poorly. When used to induce active EAE in B6 mice, MOG_37–46_ led to similar disease as MOG_35–55_-immunized mice. Using MOG_37–50_/H-2D^b^ tetramers, MOG-specific CD8^+^ T-cells were found to persist within the CNS.

While these studies utilized myelin-components to examine the potential pathogenic role of CD8^+^ T-cells in EAE, non-myelin antigen-driven systems have been used as well. One report describes CD8^+^ TCR transgenic mice recognizing glial fibrillary acidic protein (GFAP), an intermediate filament protein expressed in the CNS by astrocytes and in various peripheral tissues (17). BG1 transgenic mice are reactive to the GFAP_264–274_ peptide presented on H-2K^b^, and develop spontaneous inflammatory CNS disease by 6–12 months of age. Interestingly, GFAP-expressing vaccinia induced distinct disease pathology compared to spontaneous disease. Lesion localization and clinical manifestations of disease was dependent upon how CNS-reactive CD8^+^ T-cells were activated. CD8^+^ T-cells isolated from brains of WT BG1 mice were poor secretors of IFNγ, IL-17A, and granzyme B, suggesting alternative effector mechanisms.

Efforts to study the role of Src homology 2 domain-containing protein tyrosine phosphatase (SHP-2) in EAE demonstrated that disease could be ameliorated through phosphatase inhibition (18). The competitive inhibitor, NSC-87877 led to reduced demyelination and blocked CD8^+^ but not CD4^+^ T-cell migration into the CNS, suggesting a pathogenic role for CD8^+^ T-cells in this model.

A study of engineered transgenic NOD mice expressing a MOG_35–55_-reactive TCR (1C6) lends further support for pathogenic CD8^+^ T-cells in EAE (19). 1C6 mice spontaneously generated MOG-specific CD4^+^ and CD8^+^ T-cells that secrete pro-inflammatory cytokines. 1C6 CD8^+^ T-cells could recognize MOG_35–55_ in the context of MHC class I and II, and when adoptively transferred into NOD. Scid recipients, induced optic neuritis and mild EAE, while 1C6 CD4^+^ T-cells induced severe EAE.

CD8^+^ T-cells’ ability to target CNS components has also been evaluated in several viral models (20–22). LCMV GP33 peptide-specific CD8^+^ T-cells can induce lesions in cultured murine neurons presenting GP33 in MHC class I. While this report relies on peptide pulsing and artificial upregulation of MHC class I, viral infection-induced upregulation of class I has been demonstrated in Borna disease virus-infected rat neuronal cultures, which could be targeted by antiviral CD8^+^ T-cells, eventually leading to apoptosis of neurons (23). Although electrical signals were not initially disrupted in this model and longer incubation times were needed for neuronal apoptosis, another study has demonstrated impaired murine neuronal signaling following neuron/CD8^+^ T-cell interactions along with eventual apoptosis which interestingly occurred independent of perforin/granzymes (24). To this end, IFNγ-production from CNS CD8^+^ T-cells and subsequent IFNγ signaling in neurons has been shown to be significant for intracranial LCMV disease in mice (25). In another study, OT-I CD8^+^ T-cells formed immune synapses with MHC class I (H-2K^b^)-expressing axons presenting SIINFEKL peptide, and loss of axon integrity was observed. Additionally, axonal injury was dependent upon antigen-specific TCR recognition and granzyme B (26).

Another report also described mice expressing neo-self antigen in oligodendrocytes (ODCs) targeted by transgenic CD8^+^ T-cells (27). In this model, ovalbumin was expressed exclusively in the cytosol of ODCs and therefore ignored by CD4^+^ T-cells and B-cells. Following immunization, mild EAE was observed in some ODC–OVA mice. Studies using double transgenic ODC–OVA/OT-I mice demonstrate treatment with D1 mAb (specific for H-2K^b^/OVA) prevented the lethal EAE normally observed in these animals (28). Double transgenic mice were also given D1 prophylactically, which in certain instances led to spontaneous disease remission.

CD8^+^ T-cells have also been shown to indirectly influence CNS autoimmunity. Tc17 cells, coined for their ability to produce IL-17A, were detected in the lymph nodes and CNS of MOG_37–50_ EAE mice (29). Tc17s differ from conventional CD8^+^ T-cells regarding granzyme B and IFNγ expression, and thus are impaired in their cytotoxic capacity. In a separate study implementing CD4^+^ and CD8^+^ T-cell co-transfer, Tc17 cells were found to help CD4^+^ Th17 cells accumulate in the CNS and induce EAE (30). Furthermore, their ability to produce IL-17A was required to render CD4^+^ T-cells encephalitogenic.

## Regulatory Role for CD8^+^ T-Cells in EAE

While evidence exists to suggest a pathogenic role for CD8^+^ T-cells in MS and EAE (reviewed in Ref. (31) and discussed above), there is a growing body of evidence supporting the opposite conclusion – CD8^+^ T-cells play an important regulatory role in the pathogenesis of MS and MS-like disease. Ultimately, CD8^+^ T-cell subsets likely perform varying effector functions in the context of MS/EAE. However, the seeming discrepancy is in part due to a lack of concrete *in vivo* evidence demonstrating a cytotoxic effect of CD8^+^ T-cells in MS lesions. Furthermore, it has been demonstrated that depletion of CD8^+^ T-cells prior to EAE induction results in exacerbated disease (32). Similar results are seen in mice lacking MHC class I (although a role for NK cells can be argued) (33) and in CD8-deficient mice (32, 34, 35). This is in addition to work from our lab, which clearly demonstrated – in marked contrast to their CD4^+^ counterparts – neuroantigen-specific CD8^+^ T-cells failed to adoptively transfer EAE disease to naïve recipient mice (36). We have seen this protective CD8^+^ T-cells phenotype very robustly in several models of EAE (37).

The notion of a regulatory CD8^+^ T-cell subset (CD8^+^ Tregs) in MS is not a new idea. Studies spanning several decades point to the suppressive potential of CD8^+^ T-cells in MS patients (5–8, 38–41). In lieu of these examples, T-cell-mediated tolerance studies have largely focused on CD4^+^CD25^+^Foxp3^+^ T-cells. Although full appreciation of CD8^+^ Treg function and significance in MS and EAE is lacking, the last 15 years have seen a steady growth toward this understanding.

CD8^+^ T-cells’ suppressive ability has been described in many mouse models, including cancer (42), diabetes (43), colitis (44), SLE-like disease (45), Grave’s disease (46), and transplant tolerance (47). Inhibitory CD8^+^ T-cell subsets involved in autoimmunity in both mice and humans have been exhaustively reviewed in Ref. (48). These regulatory CD8^+^ T-cells have been extensively studied in T1D where it has been shown that low-avidity autoreactive CD8^+^ T-cells convert into memory-like autoregulatory cells and blunt diabetes progression (49, 50). However, CD8^+^ Treg participation in EAE is less-widely studied. Moreover, unlike murine CD4^+^Foxp3^+^ Tregs, a universal CD8^+^ Treg phenotype has yet to be described. For example, in EAE, CD8^+^CD28^−^ T-cells have been shown to play an inhibitory role (32) while others show CD8^+^CD122^+^ T-cells to be protective (51–53). Little is known concerning the induction of these cells in MS-like disease, though the involvement of one subtype versus another surely is influenced by disease setting and may depend on the cell’s antigen specificity/MHC-restriction. Studies of anterior chamber-associated immune deviation (ACAID) represent some of the best efforts to understand antigen-specific CD8^+^ Tregs, which appear to be Qa-1-restricted (54–56). Several ACAID studies further complicate the CD8^+^ Treg phenotyping picture (e.g., Foxp3^+^, CD94^+^, CD103^+^, TGFβ-producing, etc.) (56–60). Interestingly, immune deviation can be elicited against myelin antigens (61, 62), pointing to the potential role for Qa-1-restricted CD8^+^ T-cells in EAE disease. Qa-1-restricted CD8^+^ T-cells have been described as being important for protection in MBP-driven EAE (63). We have demonstrated that Qa-1-restricted CD8^+^ T-cells suppress EAE. We have also demonstrated that GA treatment induces CD8^+^ Treg in mice, and that these CD8^+^ T-cells are required for GA to be therapeutically effective in ameliorating EAE disease (64).

While little is still known about Qa-1-restricted CD8^+^ Tregs, even less was understood about CNS-specific CD8^+^ T-cells until very recently. We observed the surprising result that neuroantigen-specific CD8^+^ T-cells could suppress EAE induction and even ameliorate established EAE disease (36). To our knowledge, this was the first documentation of neuroantigen-specific CD8^+^ Tregs in mice. In our recently published and unpublished results, adoptive transfer of both MOG_35–55_- and PLP_178–191_-specific CD8^+^ T-cells can suppress EAE (34, 65). Due to mechanistic studies, we will elaborate upon later that these cells are quite distinct from previously described Qa-1-restricted CD8^+^ Tregs (37).

Recent work has suggested a role of IL-10-producing CD8^+^ T-cells in diminishing disease pathology in virus-induced encephalitis models. These IL-10-producing CD8^+^ T-cells display a more functional profile including increased expression of pro-inflammatory cytokines and chemokines, are immunosuppressive, and their presence in the CNS following Coronavirus infection reduces tissue destruction and morbidity in these mice (66).

## Interactions Between CD8^+^ Tregs and Other Cell Types in EAE/MS

Advancement in therapy for MS patients, particularly cellular immunotherapy, necessitates the full understanding of regulatory immune cell interplay. Studies concerning the functional interactions between CD8^+^ Tregs and other cells in the context of MS and MS-like disease are therefore of paramount interest. The next several sections will provide mechanistic insights into CD8^+^ T-cell-mediated modulation of other immune cells including CD4^+^ T-cells and antigen presenting cell (APC) populations.

### Influence of CD8^+^ T Regulatory Cells on CD4^+^ T-Cells

Qa-1-restricted CD8^+^ T-cells have been shown to modulate EAE disease through action on CD4^+^ T-cells. It has been demonstrated in a model of MBP-driven EAE that CD4^+^ T-cell vaccination protocol-mediated protection against EAE disease is dependent on the presence of Qa-1-restricted CD8^+^ T-cells that recognize specific TCRVβ molecules on MBP-reactive CD4^+^ T-cells (63). In this particular example, CD8^+^ T-cells mediated their control by preferentially suppressing Th1 CD4^+^ T-cells during EAE. While this report did not directly test cytotoxic killing as a means of suppression, the group had previously established this capability in T-cell vaccination scenarios. Data from another group later confirmed a cytotoxic effect by demonstrating that CD8αα^+^TCRαβ^+^ T-cells from lines that recognize TCRVβ8.2^+^ (MBP-reactive) CD4^+^ T-cells could protect against EAE disease in recipient mice by the targeted killing of these pathogenic cells via Qa-1-recognition (67).

We have showed that the disease-ameliorating effect of GA-therapy in EAE is dependent upon Qa-1-restricted CD8^+^ Tregs (64). In this report, we demonstrated that the protective ability of CD8^+^ T-cells was completely lost or diminished when unable to produce IFNγ or perforin, respectively. These CD8^+^ T-cells could kill GA-loaded target T-cells and even limited the proliferation of *ex vivo* neuroantigen-specific CD4^+^ T-cells (64). Furthermore, the GA-induced Qa-1-restricted CD8^+^ T-cells in this study were important for generation of CD4^+^ Tregs (64). These GA-specific CD8^+^ T-cells have the potential to kill GA-expressing CD4^+^ T-cells and limit proliferation of neuroantigen-specific and anti-CD3-stimulated CD4^+^ T-cells (8, 40). We have also demonstrated that GA therapy, whose effects require CD8^+^ T-cells in mice (64), was able to increase the induction of CD4^+^CD25^+^ Tregs from the CD4^+^CD25^−^ T-cell population in MS patient blood (40).

Distinct from the non-classical HLA-E-like Qa-1-restricted murine CD8^+^ Tregs, we have also demonstrated the existence of neuroantigen-specific CD8^+^ Tregs in MS and EAE. Neuroantigen-specific, MHC class Ia-restricted CD8^+^ T-cells can kill MOG-loaded CD4^+^ T-cells in mice (34, 36) and mediate their disease-ameliorating effects via the targeting of encephalitogenic CD4^+^ T-cells during EAE disease (34). We have also demonstrated an ability of neuroantigen-specific CD8^+^ Tregs to induce anti-inflammatory profiles in CD4^+^ T-cells during EAE (65). Importantly, we have also shown that neuroantigen-specific CD8^+^ T-cells are detectable in MS patient blood, and possess capacity to suppress CD4^+^ T-cell proliferation (5, 68).

### Influence of CD8^+^ Tregs on Dendritic Cells

The potential for CD8^+^ T-cells to alter CD4^+^ T-cell priming through direct effects on DCs is worth investigation. CD8^+^CD28^−^ T-cells have been implicated as regulators of EAE disease. It has been demonstrated that DCs have reduced costimulatory molecule (CD80, CD86, and CD40) expression after culture with CD8^+^CD28^−^ T regulatory cells, rendering these DCs as substandard APCs (32). It has been similarly demonstrated that DCs cultured with CD8^+^CD122^+^ T-cells had a reduction in CD80/86 and MHC molecules and showed inferior antigen-presentation ability compared to DCs cultured with CD8^+^CD122^−^ T-cells (52).

While it remains unclear whether Qa-1-restricted CD8^+^ Tregs have a direct effect on DCs, we have shown that neuroantigen-specific CD8^+^ Tregs can both kill and suppress antigen presentation of MOG-loaded bulk APCs (contains DCs) (36). Interestingly, we have demonstrated that neuroantigen-specific CD8^+^ Tregs have little effect on DC surface expression of MHC or costimulatory molecules, but rather shift the inflammatory profiles of CD11c^+^ DCs from IL-12 to IL-10 (65). Early human MS work from our lab points to the potential of GA therapy-induced CD8^+^ Treg-mediated killing of APCs, as CD4^+^ T-cells were only a part of the larger target pool (8).

### Influence of CD8^+^ Tregs on Monocytes/Macrophages

Another potential mechanism of suppression is CD8^+^ T-cell-mediated regulation of monocytes or macrophages, which are present in MS lesions and important for pathology in the CNS of EAE mice. Interestingly, GA treatment has been demonstrated to affect monocyte populations in EAE. For example, anti-inflammatory type II monocytes are induced in GA-treated mice, which can shift inflammatory cytokine profiles toward immunosuppressive IL-10, expand Th2 cells, and induce CD4^+^ Tregs capable of ameliorating EAE (69). We have observed similar results and have further demonstrated that the action of GA on monocytes elicits CD8^+^ Tregs and actually requires CD8^+^ T-cells for its ameliorative effects in EAE (64). This GA-induced monocyte-CD8^+^ T-cell interaction is largely unknown in MS, as is the effect of GA-induced CD8^+^ T-cell targeting of other macrophage populations. While a direct link to CD8^+^ T-cells has yet to be confirmed, studies from us and others have shown modulation of monocytes following GA therapy in humans (40, 70, 71). As mentioned in the section above, GA-induced CD8^+^ T-cell-mediated killing of APC populations like dendritic cells and monocytes/macrophages while unconfirmed, cannot be ruled out, as CD4^+^ T cells were only a portion of a larger affected target pool (8). Refining these assays for direct detection of killed targets is needed going forward.

Beyond GA-induced CD8^+^ Tregs, neuroantigen-specific CD8^+^ Tregs could conceivably modulate monocytes/macrophages in EAE. We have demonstrated that these cells can kill MOG-loaded bulk APCs, which may contain monocytes/macrophages, and can suppress their antigen presentation (36). However, we did not observe a substantial neuroantigen-specific CD8^+^ Treg effect on monocytes during EAE (65). Furthermore, neuroantigen-specific CD8^+^ Tregs from MS patients do not appear to specifically target monocytes. More work is needed to understand the potential functional interactions between CD8^+^ T-cells and monocytes/macrophages during MS and MS-like disease, and may ultimately be a GA treatment-specific phenomenon.

### Potential CD8^+^ T-Cell: B-Cell Interactions in MS/EAE?

In light of depletion therapy success, more focus is now being given to B-cells and their role in MS. The literature supports both a pathogenic (72–82) and regulatory (76, 83–94) role for B-cells in MS/EAE, and it is intriguing to speculate about the potential immune cell interplay between CD4^+^ T-cells, B-cells, and CD8^+^ T-cells therein. There is evidence in the literature to support a B-cell effect on CD8^+^ T-cells (54, 55, 82, 95–100). Many of these reports point to B-cell antigen presentation to CD8^+^ T-cells and even a B-cell requirement for CD8^+^ Treg function in some models. There is also literature supporting a role for Bregs in controlling CD8^+^ T-cell responses (17, 101–105). Additionally, CD8^+^ T-cells can be detected in follicles and modulate B-cell biology, such as germinal centers and antibody production (45, 106–111). The significance of these CD8^+^ T cell and B-cell subset interactions in the context of MS/EAE remains to be seen.

## Pathogenic Role for CD8^+^ T-Cells in MS

Due to the inherent complexity of studying CD8^+^ T-cell function in the human brain, only circumstantial evidence exists regarding a pathogenic role for CD8^+^ T-cells in MS.

CD8^+^ T-cells are the most abundant T-cells found in the CNS lesions of MS patients, far outnumbering CD4^+^ T-cells (1). In patients with active disease, CD8^+^ T-cells were detected in increasing amounts from the center to the edge of the lesions studied (112). The CD8/CD4 ratio is shown to have been as high as 50/1 in the lymphocytic perivascular cuffs at the edge of active plaques (113). CD8^+^ T-cells displaying activated and memory phenotypes (suggesting previous interaction with local antigens) have also been detected in the CNS and CSF of MS patients (3, 114). CD8^+^ T-cell clones have also been shown to move throughout the affected CNS and into normal appearing white matter (NAWM) (112). One study demonstrated that there is diffuse infiltration by CD8^+^ T-cells combined with microglial activation and meningeal inflammation in the NAWM of MS patients (115).

Unfortunately, assigning function to these CD8^+^ T-cells remains a challenging task, although speculations have been made that CD8^+^ T-cells present in the CNS lesions of MS patients may be cytotoxic toward CNS cells including glia and axons. CD8^+^ MHC class I-restricted myelin peptide-specific T-cells have been shown to cause injury to human ODCs *in vitro* (116). Similarly, an MBP-specific memory phenotype CD8^+^ T-cell line generated from the peripheral blood of MS patients, in addition to secreting IFNγ and TNFα, was able to lyse COS-MBP/HLA-A2-transfected cells that were presenting endogenous MBP (114).

CD8^+^ T-cells have also been detected near or attached to ODCs and demyelinated axons in MS patients (117–119). Importantly, MHC class I molecules are present on astrocytes, ODCs, neurons, and endothelial cells (120, 121). Furthermore, MHC class I molecules are upregulated – depending on disease severity – and can be induced by IFNγ (121). CNS blood vessel endothelium as well as several APCs also express MHC class I molecules, which can cross-present exogenous peptides (122). Thus, it is not surprising that CD8^+^ T-cells have been demonstrated to interact with APCs at CNS plaque margins (119). The potentially detrimental nature of this interaction is supported by a study that showed that the amount of CD8^+^ T-cells and macrophages present in an MS lesion is proportional to the amount of acute axonal damage present (123).

Effector cytokines from CD8^+^ T-cells can also enhance their cytotoxic function and activate other immune cells to amplify inflammatory cascades in the CNS. For example, neuroantigen-specific CD8^+^ T-cells present in the peripheral blood express IFNγ and TNFα in response to their cognate antigen *ex vivo* (6, 124, 125). IFNγ- and IL-17-producing CD8^+^ T-cells can be recruited into the CNS when responding to apoptotic T-cell-associated self-epitopes (126). One report demonstrated that CD8^+^ but not CD4^+^ T-cells from patients with acute RRMS had increased ability to be recruited in inflamed CNS venules (127). Additionally, CD8^+^ IL-17-secreting T-cell numbers have been shown to be significantly elevated in acute CNS lesions of MS patients (128). IFNγ- and IL-17-secreting CD8^+^CD161^+^ T-cells were also found to be elevated in the peripheral blood of MS patients (129). Higher frequency of CD8^+^ T-cells expressing cytotoxic molecules like perforin has been shown to be present in MS patients, particularly during a relapse (130).

## Regulatory Role for CD8^+^ T-Cells in MS

In light of the present literature, it can be appreciated that CD8^+^ T-cells in MS and other autoimmune diseases are phenotypically and functionally diverse, and can potentially regulate the pathogenic immune processes. Besides cytolytic molecules like perforin and granzyme, CD8^+^ T-cells are armed with immunosuppressive cytokines, such as IL-10, that can dampen the inflammatory response.

The evidence for CD8^+^ T-cell regulatory function in MS has existed for a long time and has been largely ignored by the field. CD8^+^ T-cells from the peripheral blood of MS patients displaying reduced levels of suppressor function was the first report that suggested a regulatory function for CD8^+^ T-cells in MS (131). This was followed by another study that demonstrated a similar defect in CD8^+^ T-cell-mediated suppression in patients with chronic progressive MS (132). Since then, mounting evidence has accumulated in the field of MS disease and others that collectively points toward a regulatory role for CD8^+^ T-cells in autoimmune diseases (50, 133, 134). More recently, our lab has provided direct evidence for CD8^+^ T-cell regulatory function in MS and has established clinical correlations with the disease activity (31).

As in the mouse, phenotypic identification of human CD8^+^ Tregs has been challenging. Human CD8^+^CD28^−^ T-cells have been shown to possess suppressor activity and are the most extensively studied population of CD8^+^ Tregs. In MS, they were found to be present at significantly reduced frequency in the blood of RRMS patients as compared to healthy donors (135). Although it is not a marker for CD8^+^ Tregs, FoxP3-expressing CD8^+^ T-cells are present in human blood. They possess regulatory activity (136), which is Foxp3-dependent (137), and are associated with autoimmune diseases such as IBD and MS (133, 138). CD8^+^FoxP3^+^ cells are present at reduced levels in the CSF of MS patients during acute exacerbation (137). CD8^+^CXCR3^+^ T-cells are human counterparts of the well-known regulatory CD8^+^CD122^+^ T-cells found in mouse. Human CD8^+^CXCR3^+^ T-cells are suppressive in nature and their function is IL-10-dependent (139). Although all of these CD8^+^ Treg subsets have potent immunosuppressive functions, so far their antigen specificity remains unknown.

Our lab showed for the first time that CNS-specific CD8^+^ T-cells have potent suppressor activity toward myelin antigen-specific CD4^+^ T-cells (5). These CNS-specific CD8^+^ T-cells were reactive to several myelin antigens including MOG, PLP, MBP, MAG and others and are present in the peripheral blood of healthy donors and MS patients (6). Mechanistically, these CNS-specific CD8^+^ T-cells are MHC class I-restricted, and their suppressive function is IFNγ-and perforin-dependent (5, 68). Our findings lend credence to the hypothesis that CNS-specific CD8^+^ T-cells in the CNS would function to dampen the inflammatory response by targeting pathogenic CD4^+^ T-cells and APCs, rather than causing damage themselves. Phenotypically, these cells are CD8^+^CD27^−^CD28^−^CD45RO^−^CD62L^−^CD57^+^ or a terminally differentiated subset of CD8^+^ T-cells (68).

Similar to Qa1-restricted CD8^+^ T in murine models, HLA-E-restricted CD8^+^ T-cells in humans perform a regulatory function and are involved in the maintenance of self-tolerance (140). The nature of NKG2 receptors present on CD8^+^ T-cells determines the functional outcome of their interaction with Qa1-expressing T-cell targets. For example, NKG2C-expressing CD8^+^ T-cells suppress Qa1-expressing target T-cells while NKG2A-expressing CD8^+^ T-cells get suppressed by these targets, and therefore cannot perform regulatory functions. A recent study showed reduced expression of FoxP3 and CD122 in NKG2C-expressing CD8^+^ T-cells from MS patients compared to healthy controls, suggesting a reduced regulatory potential of these cells in MS patients (41).

Although, there are only a handful of studies that report the phenotypic and functional significance of CD8^+^ T-cells in MS patients, one prominent feature that emerges from these studies is an underlying defect in the CD8^+^ Treg component. Of note, this defect is found specifically during MS relapses. Since a relapse represents the active phase of the disease, any significant differences in the phenotype and functions of immune cells between relapse and remission may be directly correlated with the immunopathogenesis of MS. Interestingly, frequency of circulating CD8^+^FoxP3^+^ T-cells was found to be significantly lower in the peripheral blood of MS patients during relapse as compared to remission (138). Another study showed that CD8^+^CD25^+^CD28^−^ T-cells harbored potent suppressive activity and were lower in MS patients during relapse when compared to healthy controls (141). Importantly, treatment with glucocorticoids leads to a significant increase in the frequency of these CD8^+^ Tregs in the blood of MS patients. This was an interesting observation, suggesting that recovery from relapse under glucocorticoid treatment might be mediated by the regulatory function of CD8^+^ T-cells. Furthermore, deficiency in CD8^+^ Treg function is not limited to the blood, as evidenced by the significantly reduced CD8^+^ T-cell cloning frequency in the CSF during MS relapse as compared to remission, suggesting loss of CD8^+^ Tregs in the CSF during relapse (142).

Our own studies show that the terminally differentiated CD8^+^ T-cell pool, which harbors the CNS-specific CD8^+^ Tregs, is significantly reduced during MS relapse as compared to remission (68). Furthermore, relapses in MS are associated with significantly lower CNS-specific CD8^+^ T-cell suppressor ability, while this potential in MS patients during quiescence is similar to healthy donors, suggesting a role with disease activity (5). Of clinical significance, we showed that the CNS-specific CD8^+^ Treg suppressive function is restored in MS patients during remission and this recovery in CD8^+^ Treg-mediated suppression correlated with the distance in time from an acute clinical episode. This suggests that the correction of the neuroantigen-specific CD8^+^ suppressor deficit would correlate with recovery from an acute relapse (5). One caveat to the study is that the quiescence samples could still potentially have pseudo relapses in the CNS in the absence of any clinical signs. Nonetheless, these findings raise the possibility that reduction in CNS-specific CD8^+^ T-cell suppression might be used as a marker to predict relapses in MS patients.

Although etiology of MS remains unknown, epidemiological studies suggest an association between Epstein–Barr virus (EBV) and MS (143). EBV-reactive CD8^+^ T-cells are present in the peripheral blood of MS patients (144). By using high throughput sequencing, a recent study demonstrated intrathecal enrichment of EBV-reactive CD8^+^ T-cells in MS patients (145). However, the function of these CD8^+^ T-cells in the CNS remains speculative. Interestingly, adoptive immunotherapy with *in vitro*-expanded autologous EBV-specific CD8^+^ T-cells in secondary progressive MS had no adverse effects and was associated with clinical improvement and reduced disease activity on MRI (146). This study suggests that the EBV-specific CD8^+^ T-cells in the CNS of MS patients might be playing a regulatory role by limiting EBV-infected B-cells and antibody production.

The pathogenic function of CD8^+^ T-cells in MS is believed to be largely derived from its cytotoxic potential toward CNS tissues including glial cells and axons. However, there is a clear lack of evidence in this area in human MS. Interestingly, a recent study demonstrated that CD4^+^ but not CD8^+^ T-cells from peripheral blood of MS patients expressed NKG2C and had elevated levels of cytotoxic molecules FasL, granzyme B, and perforin. Intriguingly, these CD4^+^ T-cells were cytotoxic toward HLA-E-positive human ODCs *in vitro* (147). This study suggested a novel mechanism for CNS damage in MS which is, in contrast to the widely held view, potentially mediated by CD4^+^ T-cells.

Although the pathogenic role of CD8^+^ T-cells in MS remains largely speculative, the studies discussed above strongly suggest that there is now ample evidence for the regulatory role for CD8^+^ T-cell subsets in the disease process. Lack of their regulatory function specifically during relapses should be probed further, as this could be a major underlying factor leading to relapse in MS.

## Therapeutic Induction of CD8^+^ Tregs

The majority of drugs used for the long-term management of MS are immunomodulatory in nature. The precise mechanisms by which these drugs act are under constant investigation. We have convincingly demonstrated that CD8^+^ Tregs not only exist physiologically but can also be induced therapeutically by GA treatment. Both, CD4^+^ and CD8^+^ T-cells reactive to GA are present in the peripheral blood of healthy donors and MS patients (7). Although CD4^+^ T-cell responses are comparable between the two groups, untreated MS patients have reduced GA-induced CD8^+^ T-cell responses and this deficiency is corrected after GA therapy (7). Functionally, these GA-reactive CD8^+^ T-cells are HLA-E-restricted and have a strong suppressive potential against CD4^+^ T-cells (8). Interestingly, GA-reactive CD8^+^ T-cells obtained from untreated MS patients have reduced suppressor ability and GA therapy restores the CD8^+^ T-cell suppressive potential in MS patients (8). These were the pioneering findings that linked the regulatory function of CD8^+^ T-cells with the therapeutic action of the drug. The proof of principle came from our EAE studies discussed above where we showed that GA does not work in the absence of CD8^+^ T-cells in mice (64), suggesting that CD8^+^ T-cells are absolutely required for GA action and all the other reported immunomodulatory effects of GA might lie downstream to the induction of CD8^+^ Tregs by the drug. The idea is also supported by our surprising observation that GA reverses the CD4/CD8 T-cell ratio and increases CD8^+^ T-cell-mediated suppression as early as 12 h after GA therapy initiation in humans (40). Similar to our findings, a 1-year follow-up study after IFNβ treatment showed expansion of regulatory CD8^+^ T-cell subsets (CD8^+^CD25^+^ and CD8^+^CD25^+^CD28^−^) in the responder cohort (148). Another study found a higher frequency of regulatory CXCR3^+^CD8^+^ T-cells 6 months after IFNβ therapy (149). Collectively, these studies suggest that therapeutic induction of CD8^+^ Tregs might be the underlying factor in other MS therapies as well. Natalizumab treatment results in a decreased CD4^+^/CD8^+^ ratio in the CSF and peripheral blood of MS patients (150). Fingolimod therapy is associated with altering the cytokine status of CD8^+^ T-cells in peripheral blood (151). However, detailed dissection of the role of CD8^+^ T-cells has not been performed in the setting of these treatments.

## Summary and Model

**Figure 1:**
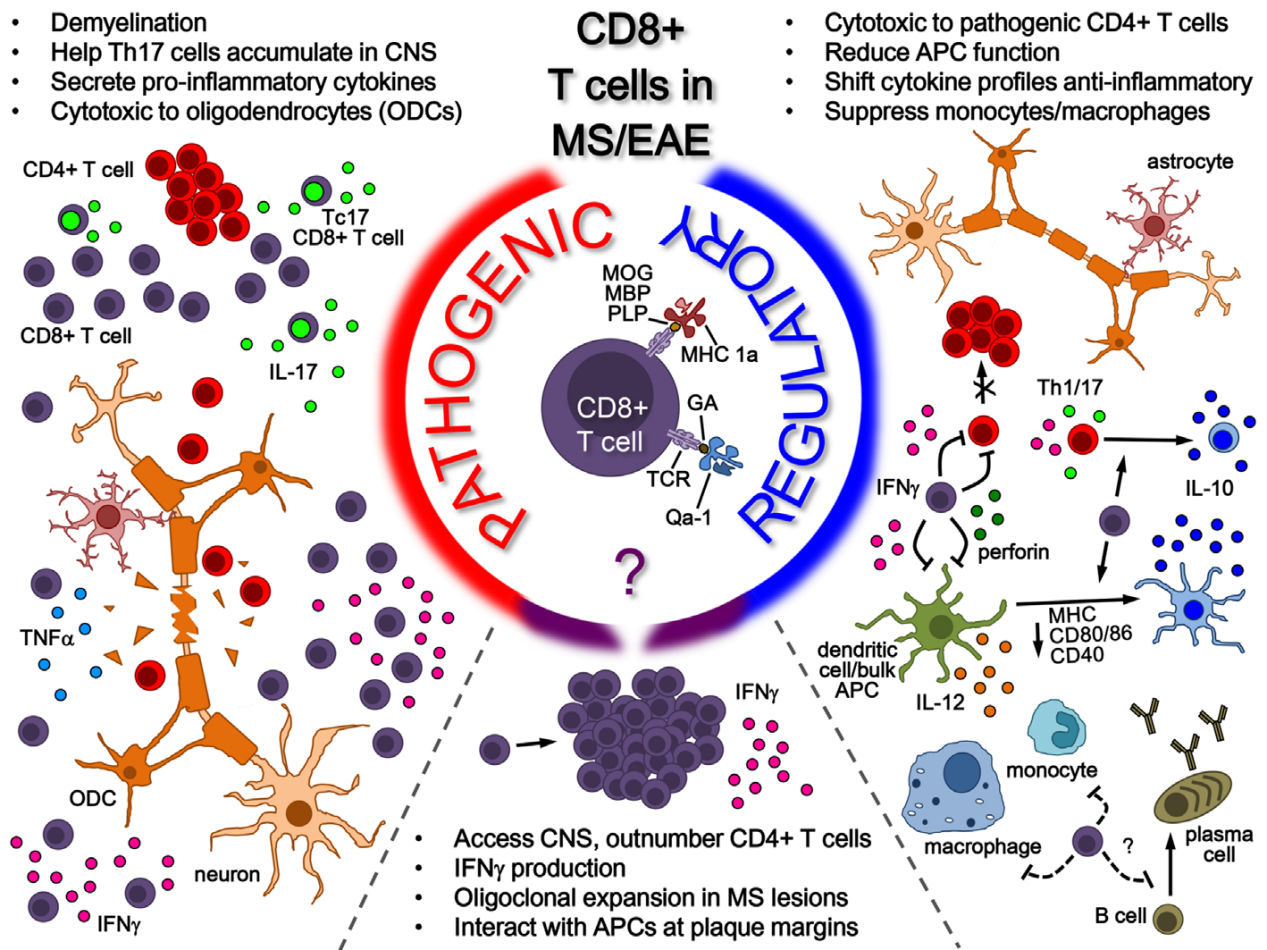
**Role of CD8^+^ T-cells in MS/EAE**. While the antigenic specificity of pathogenic CD8^+^ T-cells remains unknown, their pathogenic function is mainly attributed to pro-inflammatory cytokine secretion (in the peripheral immune system and potentially in the CNS) as well as cytotoxicity toward oligodendrocytes in the CNS. On the other side, several lines of evidence indicate a regulatory role for CD8^+^ T-cells in both MS and EAE. Neuroantigen-specific autoregulatory T-cells are classically MHC Class I restricted, whereas there are also examples of HLA-E/Qa1-restricted regulatory T-cells that may be naturally occurring or induced through therapy. Mechanisms for CD8^+^ T-cell-mediated regulation include secretion of cytokines such as IL-10 and IFNγ, cytotoxicity toward pathogenic immune cells and modulation of APC functions, both in the periphery and possibly in the CNS.

The potential roles of CD8^+^ T-cells in MS is summarized in the model shown in Figure 1, where the various pieces of evidence supporting the potential of CD8^+^ T-cells for both pathogenic and regulatory roles in MS/EAE disease are depicted. On the pathogenic side of the model, CD8^+^ T-cells, whose antigenic specificity has yet to be fully elucidated, have been shown to be involved in several disease-driving mechanisms, ranging from cytotoxicity and demyelination to pro-inflammatory cytokine production. This is in addition to hallmark activation behavior in disease lesions, such as oligoclonal expansion and IFNγ production. Interestingly, this fails to rule out the activation of a regulatory population, as indicated in the bottom portion of the model. As illustrated on the regulatory side – to which our lab has made several novel contributions – several lines of evidence exist demonstrating the regulatory mechanisms performed by CD8^+^ T-cells in the context of MS/EAE, which can either be neuroantigen specific (MHC class 1a-restricted) or GA/Copaxone^®^ specific (HLA-E/Qa-1-restricted). Their protective functions, which seem to depend on IFNγ and perforin production, range from direct cytotoxicity to pathogenic CD4^+^ T-cells to modulation of pro-inflammatory cytokine profiles to inhibition of APC function. It is still unclear to what extent CD8^+^ T-cells affect other cell populations such as B-cells, but some evidence demonstrates a suppressive effect on monocytes and macrophages. These all serve to suppress CNS auto-inflammation and protect myelinated axons – effectively limiting EAE disease pathogenesis. The potential role for these CD8^+^ Tregs in ultimately modulating MS disease is of high interest.

## Conflict of Interest Statement

The authors declare that the research was conducted in the absence of any commercial or financial relationships that could be construed as a potential conflict of interest.
